# Unilaterally blocking the muscarinic receptors in the suprachiasmatic nucleus in proestrus rats prevents pre-ovulatory LH secretion and ovulation

**DOI:** 10.1186/s12958-016-0168-7

**Published:** 2016-06-16

**Authors:** Elizabeth Vieyra, Deyra A. Ramírez, Noé Lagunas, Mario Cárdenas, Roberto Chavira, Pablo Damián-Matsumura, Angélica Trujillo, Roberto Domínguez, Leticia Morales-Ledesma

**Affiliations:** Biology of Reproduction Research Unit, Physiology of Reproduction Laboratory, Facultad de Estudios Superiores Zaragoza, UNAM, AP 9-020, CP 15000 Ciudad de México, México; Instituto Nacional de Ciencias Médicas y Nutrición “Salvador Zubirán”, Ciudad de México, México; Department of Biology of Reproduction, UAM-Iztapalapa, Ciudad de México, México; Benemérita Universidad Autónoma de Puebla, Escuela de Biología, Edificio 112A Ciudad Universitaria, CP 72570 Puebla, Puebla México

**Keywords:** Suprachiasmatic nucleus, Cholinergic system, Ovulation, Progesterone, Oestradiol, LH

## Abstract

**Background:**

The suprachiasmatic nucleus (SCN) and the cholinergic system of various regions of the hypothalamus participate in the regulation of gonadotropin-releasing hormone (GnRH) and gonadotropin secretion, which are necessary for the occurrence of ovulation. In the present study, our goal was to analyse the effects of unilaterally blocking the muscarinic receptors in the SCN on ovulation and steroid secretion.

**Methods:**

Cyclic rats were randomly allotted to one of the experimental groups. Groups of 8–14 rats were anaesthetized and microinjected with 0.3 μl of saline or a solution of atropine (62.5 ng in 0.3 μl of saline) into the left or right SCN at 09.00 or 19.00 h during diestrus-1 or on the proestrus day. The rats were euthanized on the predicted day of oestrus, and evaluated ovulation and levels of progesterone and oestradiol. Other groups of 10 rats were microinjected with atropine into the left or right SCNs at 09.00 h on the proestrus day, were euthanized eight h later, and luteinizing hormone (LH) was measured.

**Results:**

At 09.00 or 19.00 h during diestrus-1, atropine microinjections into the SCNs on either side did not modify ovulation. The atropine microinjections performed at 09.00 h of proestrus into either side of the SCN blocked ovulation (right SCN: 1/9 ovulated vs. 9/10 in the saline group; left SCN: 8/14 ovulated vs. 10/10 in the saline group). The LH levels at 17.00 h in the rats that were microinjected with atropine at 09.00 h of proestrus were lower than those of the controls. In the non-ovulating atropine-treated rats, the injection of synthetic LH-releasing hormone (LHRH) restored ovulation. Atropine treatment at 19.00 h of proestrus on either side of the SCN did not modify ovulation, while the progesterone and oestradiol levels were lower.

**Conclusion:**

Based on the present results, we suggest that the cholinergic neural information arriving on either side of the SCN is necessary for the pre-ovulatory secretion of LH to induce ovulation. Additionally, the regulation of progesterone and oestradiol secretion by the cholinergic innervation of the SCN varies with the time of day, the day of the cycle, and the affected SCN.

## Background

The suprachiasmatic nucleus (SCN) of the hypothalamus contains a “central pacemaker” that orchestrates circadian rhythms. The circadian system is important for successful reproduction because it ensures that the period of maximal fertility aligns with peak of sexual motivation [1]. The SCN regulates gonadotropin-releasing hormone (GnRH) secretion, the pre-ovulatory surge of luteinizing hormone (LH) and ovulation [2–5].

The pulsatile frequency and secretion amplitude of GnRH regulate gonadotropin and sex steroid hormone secretion, follicular maturation and ovulation [6–11]. According to Robertson et al. [12], there are two possible routes by which the SCN could regulate GnRH neurons and hence the LH surge. One pathway involves the direct innervation of the GnRH neurons by the SCN. Direct synapses exist between the neurons in the SCN and GnRH neurons in the preoptic anterior area (POA), and vasoactive intestinal peptide (VIP) and vasopressin (AVP) act as neurotransmitters in these synapses [13, 14]. The second pathway involves an indirect circuit linking the SCN with the GnRH neurons through a relay station in the anteroventral periventricular nucleus (AVPV), which is a hypothalamic region that is critical for the GnRH/LH surge in rodents. The SCN sends AVPergic fibres directly to the AVPV [15] where kisspeptidergic neurons have been identified [9]. These neurons are probably responsible for generating the oestradiol-mediated GnRH and LH surges that are required for ovulation [16].

The central cholinergic system regulates the circadian system and modulates the activities of neurons in the SCN. In rats, the SCN receives cholinergic projections from the basal forebrain, including the nucleus basalis magnocellularis (NBM), the brainstem (from the pedunculopontine tegmental nucleus (PPTg)), and the latero-dorsal tegmental nucleus (LDTg) [17]. According to Yang et al. [18], multiple cholinergic-modulatory actions occur via multiple receptors and ion channels that may allow acetylcholine to exert delicate control of the excitabilities of SCN neurons in different physiological settings. Damaging the NBM with quinolic acid reduces SCN cholinergic input and decreases the expressions of VIP and AVP by SCN neurons. The extents of the declines in the expressions of VIP and AVP correlate with the numbers of destroyed cholinergic afferents, which supports the notion that acetylcholine plays a direct role in regulating the metabolic activities of SCN neurons [19].

The SCN sends a cholinergic efferent to the diagonal band of Broca and the medial septum [17, 20], and GnRH neurons are present in these two nuclei [21], which suggests the possibility that the cholinergic system of the SCN regulates GnRH neurons via a direct cholinergic pathway. According to Turi et al. [22], the GnRH neurons are regulated by cholinergic neurons located in different areas of the brain in apposition to GnRH neurons. Everett et al. [23] demonstrated that at proestrus, the cholinergic system participates in the regulation of ovarian hormone secretion and ovulation. Unilaterally implanting atropine crystals into either side of the preoptic-anterior hypothalamic area (POA-AHA) blocks ovulation in a manner that depends on the side into which the POA-AHA atropine is implanted and the day of the oestrous cycle on which the treatment is performed [24]. Moreover, GnRH injection at the predicted time of proestrus restores ovulation [25]. The participation of the muscarinic system in the regulation of progesterone and oestradiol serum levels varies with the hours and days of the oestrous cycle [26], [27].

Asymmetry is a natural trait that is displayed across the range of basic particles to complex organ functions [28]. The SCN consists of two bilaterally paired nuclei [29], each of which is capable of generating a behavioural activity rhythm [30] and may act as an independent oscillator [31]. To our knowledge, there is no information on the effects on spontaneous ovulation that are elicited by the unilateral block of the cholinergic receptors in the SCN. Since in rats on diestrus-1 the unilateral implant of atropine on either side of the POA-AHA blocked ovulation, while the same treatment with rats on proestrus day did not [24] and the subcutaneously injecting atropine sulphate to rats on each day of the oestrous cycle blocked ovulation, depending on day and hour of treatment [32], the aims of the present study were to analyse the effects of unilaterally blocking the SCN’s muscarinic system in rats during diestrus-1 or proestrus on ovulation. According to Zhao and Kriegsfeld [33] immortalized GnRH cells exhibit intrinsic daily changes in their sensitivity to neurochemicals stimulating their activity, which did not depend on clock genes. To test the possibility that blocking SCN cholinergic inputs has an effect on ovulation regulation, the effects of injecting atropine into either side of the SCN were evaluated at two different times of day, i.e., during the light period at 09.00 h and at the beginning of the dark period at 19.00 h.

## Methods

### Animals

This study was performed with adult, virgin, 3-month-old, female, CIIZ-V strain rats weighing 230–260 g from our own breeding stock. The animals were maintained under controlled light (on from 05:00 to 19:00 h) and temperature (22 ± 2 °C) conditions with free access to food (Purina S.A., México) and tap water. The animals’ oestrous cycles were monitored via cytological examinations of daily vaginal smears. Only rats that exhibited at least two consecutive 4-day oestrus cycles were used in the experiment.

### Unilateral microinjection of atropine into the left or right SCN

**Fig. 1:**
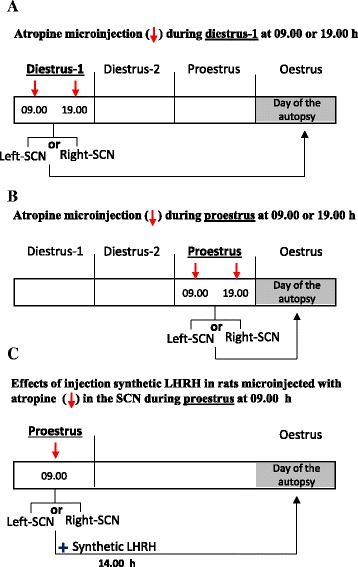
Schematic representation of the treatment design. The groups of rats were microinjected with atropine in the left or right SCN at 09.00 or 19.00 h during diestrus-1 (**a**), and the other groups were microinjected on the proestrus day (**b**). In panel **c**, note that the group of rats were microinjected with atropine in the *left* or *right* SCN at 09.00 h and at 14.00 h these rats were subcutaneously injected with 3.7 μg/kg synthetic LHRH. All animals were sacrificed on the predicted vaginal oestrus day

The experimental protocol is illustrated in Fig. 1a and b. At 09.00 or 19.00 h, groups of 4-day cyclic rats in diestrus-1 or proestrus were anaesthetized with sodium pentobarbital (30 mg/kg I.P.; Pisabental, PISA Agropecuaria S.A. de C.V. México). To assess the effectiveness of the anaesthesia, we used the tail pinch method [34]. After verifying that the rats were anaesthetized, they were placed in a stereotaxic apparatus (David Kopf Instruments, Tujunga, CA). The skin of the skull was sectioned, and the left or right side of the skull was drilled with a 1-mm bit. Subsequently, a 29-gauge stainless steel microinjection needle was lowered into the left or right side of the SCN. The SCN was located according to the coordinates of the Paxinos and Watson [35] atlas (left SCN: −0.3 mm AP, +0.2 mm ML, and −9.1 mm DV; right SCN: −0.3 mm AP, −0.4 mm ML, and −9.1 mm DV). The microinjection needle was connected to a 25-μl Hamilton syringe placed on a microinjection pump (CMA/100; BAS, Stockholm, Sweden) with a Teflon tube (0.65 mm OD 9, 0.12 mm OI; Bioanalytical Systems, Inc., West Lafayette, IN). Next, 0.3 μl of saline or 62.5 ng of atropine (based on reference [24, 25, 32]) in 0.3 μl of saline (atropine is a non-specific muscarinic blocker with a half-life of 2 h; Sigma Chemical Co., St. Louis, MO) was microinjected into the left (saline *n* = 36; atropine *n* = 42 rats) or right SCN (saline *n* = 36; atropine *n* = 35 rats). After surgery, each animal was placed in an individual cage with warm sawdust and heated with an incandescent lamp until the animal awoke.

To examine the potential diffusion of the liquid from one SCN into the other, ten rats were microinjected with 0.3 μl of methylene blue dye in saline solution (10 mg/ml) in the left or right SCN [36]. No diffusion of methylene blue dye into the contralateral SCN, the ipsilateral nuclei (including the POA), or the third ventricle was observed.

### Effects of the unilateral microinjection of atropine on the pre-ovulatory LH peak

To assess whether the atropine treatment modified the pre-ovulatory LH surge that occurs at 17.00 h on the proestrus day, groups of rats were injected at 09.00 h on the proestrus day with either vehicle or atropine in the left SCN (*n* = 10 per group) or right SCN (*n* = 10 per group) following the procedures described above. The rats were sacrificed at 17.00 h on the same day.

### Effects of injecting synthetic LH-releasing hormone on the ovulation responses of non-ovulating rats microinjected with atropine in the SCN

Following the procedures described above, groups of rats on the proestrus day were treated at 09.00 h with atropine in the left SCN (*n* = 9 per group) or right SCN (*n* = 9 per group) and treated again at 14.00 h (5 h later). The rats were subcutaneously injected with 3.7 μg/kg of synthetic LH-releasing hormone (LHRH)-Gly-OH (Sigma Chemical Co., St. Louis, MO) diluted in saline following the protocol of Humphrey and colleagues [37]. The rats were sacrificed the next day (predicted oestrus) between 09.00 and 10.00 h (Fig. 1c).

### Effects of the microinjection of atropine into the SCN at 09.00 h on the proestrus day on ovarian morphology

To assess the effects the microinjection of the atropine into the SCN on ovarian morphology, the left and right ovaries from the experimental rats were removed, cleaned of adherent fat tissue, fixed in Bouin’s solution for 24 h, dehydrated and embedded in paraffin. The ovaries (left and right) of three randomly selected rats from each group [i.e., the atropine-microinjected and vehicle-injected groups] were serially sectioned at 10 μm and stained with haematoxylin-eosin. All ovary sections were analysed for the presence of antral follicles and new and old corpora lutea with the aid of a Nikon binocular microscope.

### Autopsy procedures

On the predicted vaginal oestrus day (72 h or 24 h after treatment), the rats were sacrificed by decapitation [38, 39]. The blood of the trunk from each animal was collected, allowed to clot and centrifuged at 3,000 rpm for 15 min. The serum was stored at −20 °C until the progesterone, oestradiol and LH concentrations were measured via specific radioimmunoassays. Upon autopsy, the oviducts were dissected, and the numbers of ova shed were counted with the aid of a stereoscopic microscope (Nikon, Model C-PS). The brain was subsequently dissected and quickly placed on a plate cooled with dry ice to verify the accuracy of the microinjection site.

### Brain histologic processing

**Fig. 2:**
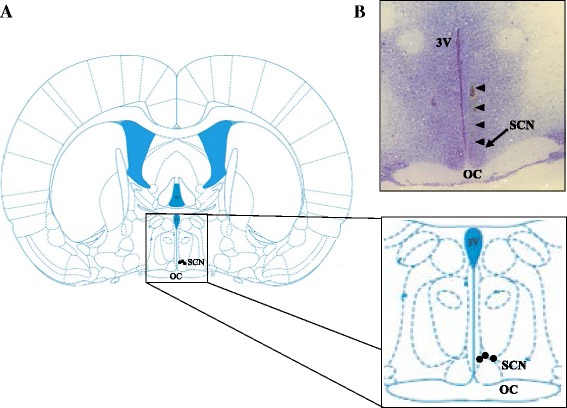
Diagrammatic representation of the locations of the microinjection sites. **a** The *black circles* represent the microinjection sites. Schematic illustration of a coronal section taken from the rat brain atlas of Paxinos and Watson [35]. **b** Nissl-stained coronal sections illustrating the trajectories of the microneedles into the left SCN. 3 V: third ventricle; OC: optic chiasm; SCN: suprachiasmatic nucleus. The *arrowheads* indicate the trajectory of the microinjections into the *left* side of the SCN

The brains of all rats treated with vehicle or atropine were frozen, and 100-μm sections were obtained on a cryostat (MICROM HM 505 N, Walldorf, Germany). The section were mounted on slides and stained with 1 % cresyl violet. All results of the present study are based on rats with verified microinjections into the SCN (Fig. 2a, b).

### Hormone measurements

The serum concentrations of progesterone (ng/ml) and oestradiol (pg/ml) were measured using radioimmunoassay (RIA) with kits purchased from Diagnostic Products (Los Angeles, CA, USA). The intra-assay coefficients of variation were 8.35 and 8.12 % for the progesterone and oestradiol assays, respectively, and the inter-assay coefficients of variation were 9.45 and 9.28 % for the progesterone and oestradiol assays, respectively.

The LH levels in the sera (ng/ml) were measured using the double antibody RIA technique with reagents and protocols kindly supplied by the NIADDK National Pituitary Program (Bethesda, MD, USA). The intra- and inter-assay variations were approximately 5.1 and 6.5 %, respectively, for LH. The results are expressed in terms of the NIADDK standards RP-2 for FSH and LH.

### Statistical analysis

The statistical analyses were performed using GraphPad Instant 3. The ovulation rates (i.e., the numbers of ovulating animals/the numbers of treated animals) were analysed using Fisher’s exact probability or Chi-square tests. Data regarding the numbers of ova shed were analysed using Kruskal–Wallis tests followed by a Mann–Whitney *U* tests. The hormonal serum level results were analysed using analysis of variance (ANOVA) followed by Tukey’s tests. When two means were compared, we used Student’s *t*-tests. *P*-values below 0.05 were considered statistically significant.

## Results

### Ovulation and the numbers of ova shed

To investigate whether the cholinergic system of the left and/or right SCN regulated spontaneous ovulation in the rat, the ovulation rates and the numbers of the ova shed were determined. The ovulation rates and the numbers of ova shed were not modified by the microinjection of atropine into either side of the SCN at 09.00 or 19.00 h during diestrus-1 (Table 1).

**Table 1:** Ovulatory responses of the rats with atropine into the SCN during diestrus-1 or proestrus. The ovulation rates and the means ± SEMs of the numbers of ova shed by the rats subjected to microinjections of the vehicle into the left SCN (Vh L-SCN) or the right SCN (Vh R-SCN) and those subjected to atropine microinjections into the left SCN (ATR L-SCN) or the right SCN (ATR R-SCN) at 09.00 or 19.00 h during diestrus-1 or proestrus

Groups	*n*	Ovulation rate^b^	Number of ova shed	*n*	Ovulation rate^b^	Number of ova shed
09.00 h of diestrus-1				19.00 h of diestrus-1		
Vh L-SCN	8	8/8	10.4 ± 2.1	10	10/10	10.1 ± 1.2
ATR L-SCN	10	10/10	11.5 ± 1.2	10	10/10	8.8 ± 1.1
Vh R-SCN	10	10/10	8.5 ± 1.0	8	7/8	10.0 ± 1.9
ATR R-SCN	10	9/10	9.5 ± 1.1	8	8/8	8.3 ± 1.3
09.00 h of proestrus				19.00 h of proestrus		
Vh L-SCN	10	10/10	8.5 ± 1.0	8	7/8	10.0 ± 1.9
ATR L-SCN	14	8/14^*^	9.8 ± 1.2	8	8/8	11.5 ± 1.4
Vh R-SCN	10	9/10	11.4 ± 1.6	8	8/8	8.3 ± 1.3
ATR R-SCN	9	1/9^**, ***^	3^a^	8	8/8	11.6 ± 0.8

^*^ *p* < 0.02 vs. the respective group treated with Vh L-SCN (Fisher’s exact probability test)

^**^ *p* < 0.001 vs. the respective group treated with Vh R-SCN (Fisher’s exact probability test)

^***^ *p* < 0.04 vs. ATR L-SCN (Fisher’s exact probability test)

^a^represents the number of ova shed by the only ovulating rat

^b^Number of ovulating animals/number of treated animals

The microinjection of atropine into the left SCN at 09:00 h during proestrus resulted in a lower ovulation rate (57 % of the atropine-treated rats ovulated vs. 100 % of the saline-injected group; *p* < 0.001, chi-square test). Only 11 % of the rats that were microinjected with atropine into the right SCN ovulated, whereas 90 % of those microinjected with saline ovulated. No differences in the numbers of ova shed by the ovulating rats that were microinjected with vehicle or atropine were observed (Table 1).

At 19.00 h during proestrus, the atropine treatments on either side of the SCN did not modify the ovulation rate or the number of ova shed (Table 1).

### Progesterone and oestradiol serum levels in the rats microinjected with atropine during diestrus-1

**Fig. 3:**
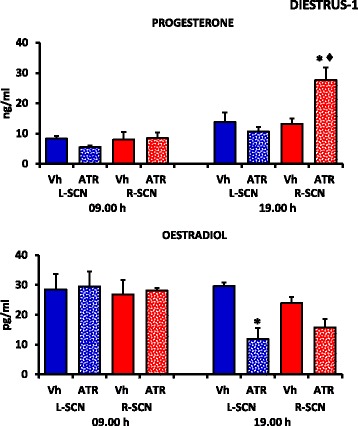
Steroid hormone serum levels in the rats treated at 09.00 and 19.00 h during diestrus-1. Means ± SEMs of the progesterone (ng/ml) and oestradiol serum levels (pg/ml) in the rats subjected to microinjections of vehicle into the left (Vh L-SCN) or right (Vh R-SCN) SCN or atropine microinjection into the left (ATR L-SCN) or right (ATR R-SCN) SCN at 09.00 or 19.00 h during diestrus-1. *p < 0.05 vs. the respective groups microinjected with Vh (ANOVA followed by Tukey’s test). ♦*p* < 0.001 vs. ATR-L-SCN (ANOVA followed by Tukey’s test)

Compared with the saline-microinjected animals, atropine microinjection at 09.00 h in either SCN did not modify the serum progesterone or oestradiol levels (Fig. 3).

In the rats that were microinjected with atropine in the right SCN at 19.00 h, the progesterone levels were higher, whereas atropine treatment in the left SCN resulted in lower oestradiol levels compared to the rats that were microinjected with the vehicle (Fig. 3). Atropine microinjection in the right SCN yielded higher progesterone levels compared with those of the rats that were treated in the left SCN (Fig. 3).

### Serum progesterone and oestradiol levels in the rats that were microinjected with atropine during proestrus

The rats in proestrus that were treated with atropine into either side of the SCN at 09.00 h exhibited significantly lower ovulation rates; consequently, the progesterone and oestradiol levels in the ovulating and non-ovulating animals were analysed separately.

**Fig. 4:**
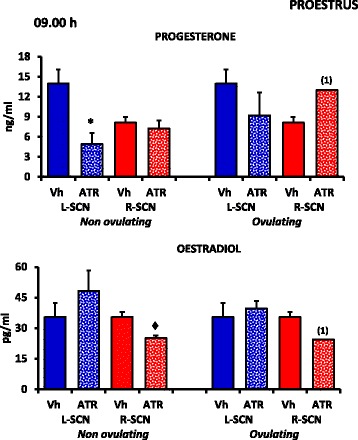
Steroid hormone serum levels of the rats treated at 09.00 h on the proestrus day. The means ± the SEMs for the progesterone (ng/ml) and oestradiol serum levels (pg/ml) of the non-ovulating and ovulating rats that received microinjections of the vehicle in the left (Vh L-SCN) or right (Vh R-SCN) SCN or atropine microinjection in the left (ATR L-SCN) or right (ATR R-SCN) SCN at 09.00 h during proestrus. (1) Represents the level of one animal’s ovulation. * *p* < 0.01 vs. the respective groups microinjected with Vh (ANOVA followed by Tukey’s test). ♦*p* < 0.05 vs. ATR-L-SCN (ANOVA followed by Tukey’s test)

Compared with the group that was microinjected with vehicle, the non-ovulating rats that were microinjected with atropine into the left SCN (*n* = 6) exhibited lower progesterone levels. Atropine microinjection into the right SCN (*n* = 8) yielded lower oestradiol levels compared with those of the rats that were treated in the left SCN (*n* = 8; Fig. 4). In the ovulating rats that were microinjected with atropine in the left SCN (*n* = 8), the serum progesterone and oestradiol levels were not different from those of the saline-microinjected rats. A statistical analysis of the difference between the ovulating rats that were treated with atropine in the right SCN and the corresponding vehicle-treated group was not possible because only one rat in the right SCN atropine treatment group ovulated (Fig. 4).

**Fig. 5:**
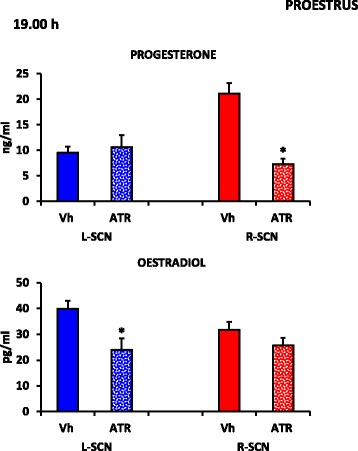
Steroid hormone serum levels of the rats treated at 19.00 h on the proestrus day. The means ± the SEMs of the progesterone (ng/ml) and oestradiol serum levels (pg/ml) in the rats subjected to microinjections of vehicle in the left (Vh L-SCN) or right (Vh R-SCN) SCN or atropine microinjections in the *left* (ATR L-SCN) or right (ATR R-SCN) SCN at 19.00 h during proestrus. * *p* < 0.05 vs. their respective groups that were microinjected with Vh (Student’s *t* *-*test)

In the proestrus rats that were treated at 19.00 h with atropine into the right SCN, the progesterone levels were lower than those in the corresponding vehicle-treated group. The atropine treatment in the left SCN resulted in lower oestradiol levels compared with those of the corresponding vehicle-treated group of rats (Fig. 5).

### LH serum levels at 17.00 h during proestrus

**Fig. 6:**
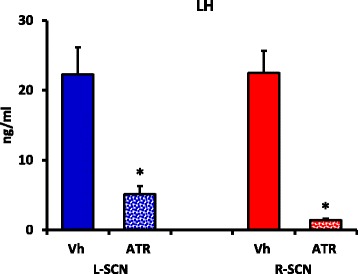
LH serum levels evaluated at 17.00 h on the proestrus day. The means ± the SEMs for the LH serum levels (ng/ml) of the rats subjected to microinjection of the vehicle in the *left* (Vh L-SCN) or *right* (Vh R-SCN) SCN or atropine microinjection into the *left* (ATR L-SCN) or *right* (ATR R-SCN) SCN at 09.00 h during proestrus and sacrificed at 17.00 h on the same day. * *p* < 0.01 vs. the respective groups that were microinjected with Vh (Student’s *t* *-*test)

The LH levels in the rats that were microinjected with atropine at 09.00 h on either side of the SCN and sacrificed at 17.00 h were lower than the LH levels the corresponding vehicle-microinjected group (Fig. 6). Atropine treatment in the right SCN yielded lower LH levels compared with those of the rats with left SCN treatments (ATR R-SCN 1.4 ± 0.2 vs. ATR L-SCN 5.1 ± 1.3; *p* < 0.023, Student’s *t-*test).

### Influence of LHRH on ovulation in the rats that were microinjected with atropine in the SCN

**Fig. 7:**
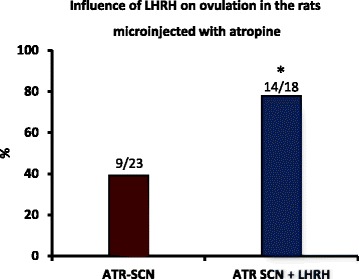
Influence of LHRH on ovulation in the rats that were microinjected with atropine. Ovulation rates of the rats subjected to microinjections of atropine in the SCN (ATR-SCN) at 09.00 h during proestrus and rats microinjected with atropine followed by injection with 3.7 μg/kg per body weight of synthetic LHRH at 14.00 h on the same day (ATR-SCN + LHRH). The animals were sacrificed 18–20 h after treatment on the predicted oestrus day. *p < 0.025 vs. the atropine group (Fisher’s exact probability test)

Because the microinjection of atropine into the SCN at 09.00 h during proestrus blocked ovulation, and the pre-ovulatory release of GnRH is required for ovulation, we examined whether the microinjection of atropine into the SCN altered the pre-ovulatory release of GnRH. The results obtained from the rats that were microinjected with atropine in either SCN were combined. Among the rats that were microinjected with atropine in the SCN, ovulation was induced in 77 % of the rats that were treated following a subcutaneous injection of synthetic LHRH (Fig. 7).

### Ovarian morphology

**Fig. 8:**
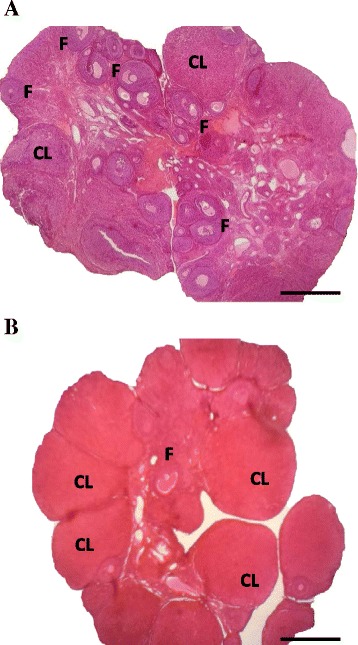
Ovarian histologies of the rats with atropine into the SCN at 09.00 h during proestrus. The micrographs correspond to the greatest section of the ovary at 10-μm thick that was stained with haematoxylin-eosin. The rats were sacrificed on the predicted vaginal oestrus day. **a** the ovaries of rats following the microinjection of the vehicle into either SCN (Vh-SCN) in which several fresh corpora lutea can be observed and some antral follicles can be observed. **b** Ovaries of rats microinjected with atropine in either SCN (ATR-SCN); F, antral follicles; CL, corpora lutea. 4X microscopic lens, Scale Bar = 200 μm

Independently of the day and time, vehicle microinjection into either side of the SCN did not modify the ovarian morphology (Fig. 8a). A histological analysis of the ovaries of the non-ovulating rats that were treated with atropine in either side of the SCN at 09.00 h during proestrus revealed the presence of a few antral follicles and old corpora lutea (Fig. 8b).

## Discussion

The present results suggest that the cholinergic neural information arriving to either side of the SCN at 09.00 h is necessary for the pre-ovulatory secretions of GnRH and LH that result in spontaneous ovulation in proestrus rats. This neural information does not appear to participate in the regulation of GnRH or LH secretion on the proestrus day at 19.00 h or during diestrus-1 at 09.00 or 19.00 h.

Funabashi et al. [40] demonstrated that the injection of pentobarbital on the morning of proestrus (8.00–09.00 h) does not interfere with the pulsatile secretion of LH. Domínguez and Smith [41] demonstrated that in 4- and 5-day cyclic rats on the proestrus day, the injection of pentobarbital at 09.00 h does not modify ovulation. A similar effect was observed in the present study. In rats on the proestrus day, the injection of barbiturates or atropine at 13.00 h blocks ovulation, and the injection GnRH, LH or human chorionic gonadotrophin (hCG) at 17.00 h (4 h later) restored ovulation, which suggests that the blocking agents (barbiturates or atropine) affect the spontaneous release of LH [41–43].

We have previously demonstrated that systemically blocking the cholinergic system via the subcutaneous injection of atropine sulphate blocks spontaneous ovulation on the predicted oestrus day in a manner that depends on the day and time of the oestrous cycle, which suggests that gonadotropin secretion throughout the oestrous cycle is controlled by a circadian rhythm and that this circadian rhythm is closely related to the cholinergic system [32].

We have previously demonstrated that the blockade of the cholinergic system via the implantation of atropine crystals at 13.00 h into either side of the POA-AHA during diestrus-1, into the right POA-AHA during oestrus, and into the left POA-AHA during diestrus-2 results in the lack of ovulation on the predicted day of oestrus. However, implantation on either side of the hypothalamus during pro-oestrus does not modify the rate of ovulation [24]. In the present study, the unilateral block at 09.00 h of the muscarinic receptors in the SCN of rats on the proestrus day partially blocked ovulation, whereas the same treatment had no effect in rats during diestrus-1. These results are the opposite of those observed when we blocked the cholinergic system in the POA-AHA region (i.e., complete blockade in diestrus-1 rats and a lack of effect in proestrus rats). Taken together, the present and previous results suggest that, during the first three days of the cycle, the POA-AHA regions participate in the regulation of GnRH secretion and ovulation, whereas the participation of the SCN’s cholinergic system occurs only during the beginning of the proestrus day.

In the adult rat, the SCN’s cholinergic afferents that originate in the NBM play an important role in maintaining the anatomy and chemo-architecture of the SCN [19, 44]. On the afternoon of proestrus, the AVP innervation arising from the SCN stimulates the pre-ovulatory LH surge [45]. Loh et al. [46] demonstrated that, in female VIP-knockout mice, the ovulatory response is reduced. In the present study, the ovulation blockade that resulted from the atropine treatment of the rats in proestrus at 09.00 h might have resulted from a decrease in the neural signals from the AVPergic and/or VIPergic neurons that regulate the GnRH neurons located in the POA. Moreover, this decrease would prevent the pre-ovulatory LH surge required for ovulation. The loss of VIP signalling can result in a decrease in reproduction [46].

According to Cruz et al. [47], blocking the muscarinic receptors in the POA-AHA during diestrus-2 or oestrus appears to be linked to the inhibition of ovarian follicular population growth in the ovary ipsilateral to the treated hypothalamic area. In another experiment, the blockade of M_1_R muscarinic receptors in the left ovary was observed to block ovulation, whereas this block had no effect when performed in the right ovary. Based in these results, the authors suggested that the M_1_R receptors in the left ovary might regulate ovulation asymmetrically through a stimulatory neural signal that is relayed to the hypothalamus via the vagus nerve and induces GnRH secretion, which then triggers ovulation [48].

Ovulation is a complex process that leads to the release of the mature oocyte from the ovarian follicle, induced by the LH surge [49]. Part of the events for LH-induced ovulation include the weakening of the follicle wall by proteolytic digestion, apoptotic follicle cell death, and follicle contraction. Overall, in mammals these coordinated events are crucial for follicle rupture and subsequent expulsion of the oocyte [50]. In proestrus day, acetylcholine binding to M_1_R in the follicular cells of the left ovary generates a neural signal involved in the regulation of GnRH secretion, which in turn, stimulates LH secretion, which is required for ovulation [48].

Although present results cannot rule out the possibility that the pre-ovulatory LH surge underwent a phase change (advance or delay), the presence of a few antral follicles and old corpora lutea in the ovaries of non-ovulating rats suggest that the low levels of LH measured at 17.00 h during proestrus were not sufficient to stimulate the final follicle maturation phase. Our interpretation is supported by the observed ovulation induced by injecting LHRH at 14.00 h to atropine treated rats during proestrus and by Cruz et al. [47] results showing that in rats on oestrus day, blocking the muscarinic receptors in the right POA-AHA resulted in a greater number of pre-ovulatory atretic follicles in the ipsilateral ovary, suggest that the lack of ovulation in rats with an atropine implant on the right POA-AHA was the result of an insufficient LH surge on proestrus. Based on these results we suggest that the cholinergic signal originating at the SCN regulates the follicle’s final maturation phase, perhaps through a cholinergic neuronal pathway.

Because the injection of synthetic LHRH into the rats that were treated with atropine in the right SCN induced ovulation, we propose that the cholinergic signal originating in the SCN is necessary for the regulation of GnRH and LH secretion. In proestrus rats, the injection of atropine into the left or right SCN at 09.00 h resulted in lower LH levels, which suggests that on the morning of proestrus, the cholinergic system innervating the SCN regulates gonadotropin secretion in a stimulatory manner. Moreover, the present results suggest that a pre-ovulatory LH surge shift was not produced. In vitro studies have demonstrated that carbachol (a nonspecific acetylcholine agonist) acts directly on the SCN to alter the phase of the rhythm of neuronal activity and that atropine and pirenzepine (a M1-selective antagonist) block carbachol’s phase-shifting effects [51]. Cruz et al. [52] reported that atropine-sulphate treated animals exhibit a 24-h delay in the LH preovulatory surge.

There is evidence that kisspeptin regulates GnRH secretion at two different levels, i.e., the kisspeptinergic signal originating from the AVPV that acts on the somata of GnRH neurons [53] and the arcuate kisspeptinergic neurons at the GnRH axons [54]. Because the SCN sends AVPergic information to both the AVPV and arcuate kisspeptinergic neurons, the present results suggest that the somata of the SCN cells that release AVP are stimulated by acetylcholine ligand binding on their muscarinic receptors. The differences in LH levels observed in the proestrus rats that were microinjected at 17.00 h with atropine into either SCN suggest that the cholinergic signals originating from the left and right SCNs modulate GnRH secretion from two different zones of the hypothalamus.

There is evidence that the regulation exerted by the hypothalamus on ovarian function exhibits presents asymmetry [55] and that this asymmetry varies during the oestrous cycle [24, 25]. Gerendai and Halász [55] suggested that the right side of the hypothalamus plays a dominant role in the control of reproductive functions. We have previously demonstrated that the right side of POA-AHA exhibits greater choline acetyltransferase activity during the day of oestrous, whereas this activity is greater in the left POA-AHAs of rats studied during diestrus-2 [56]. In the SCN, there is evidence of asymmetries, i.e., the asymmetrical patterns of activity from each side of the SCN support the hypothesis that independent oscillators exist on each side. This asymmetry could reflect a more general feature of brain organization, namely, functional lateralization [31]. According to Yang and Okamura [57] and Yan and Silver [58], the expressions of Per1 in the middle and rostral SCN regions are asymmetric. In adult male Syrian hamsters, exposure to constant light induces a splitting in their locomotor activity that results in asymmetry in the Per1 expression in the SCN [59]. In the present study, the differences in the capacities of atropine microinjections into the right and left SCNs at 09.00 h of proestrus to affect spontaneous ovulation (i.e., the ovulation rate) suggest that there are differences in the sensitivities of the muscarinic systems of each SCN. These results suggest that the right SCN seems to be more committed to the regulation of ovarian function. Although additional studies are required to clarify this idea, we cannot rule out the notion that the differences could be related to the numbers of muscarinic receptors in each SCN.

The SCN regulates the activity of the adrenal gland by acting on neurons that release corticotrophin-releasing hormone (CRH) and AVP [60, 61]. We have previously demonstrated that the adrenal glands are the main source of progesterone during the oestrous cycle in rats [26]. According to Kornya et al. [62], the cholinergic action on steroid production in human granulosa cells is mediated through the muscarinic route, and cholinergic neurotransmission may play a physiological role in intraovarian regulatory pathways. According to Cruz et al. [27], the participation of the cholinergic system in the regulation of ovarian oestradiol secretion varies throughout the oestrous cycle, and in unilaterally ovariectomized rats, this participation also depends on the in situ ovary.

In the present study, the changes in the progesterone and oestradiol levels in the rats that underwent unilateral atropine microinjection depended on the hormone measured, the time of day of the treatment, and the side of the SCN treated, which suggests that the neuroendocrine mechanisms that regulate hormone secretion exhibit asymmetry. Additionally, the regulations of progesterone and oestradiol secretions are modulated by SCN signals after pre-ovulatory LH release occurs.

## Conclusions

Based on the present results, we propose that, for ovulation to occur, the system regulating GnRH and LH secretion requires a cholinergic signal from the left or right SCN on the morning (09.00 h) of proestrus. Together, the present results suggest that the cholinergic innervation of the SCN participates in the regulation of progesterone and oestradiol secretions and that this participation varies according to the hour and day of the cycle studied and exhibits functional asymmetry.

## Abbreviations

ARC, arcuate nucleus; AVP, vasopressin; AVPV, anteroventral periventricular nucleus; GnRH, gonadotropin-releasing hormone; LDTg, latero-dorsal tegmental nucleus; LH, luteinizing hormone; NBM, nucleus basalis magnocellularis; POA, preoptical area; PPTg, pedunculopontine tegmental nucleus; RP3V, rostral periventricular area of the third ventricle; SCN, suprachiasmatic nucleus; VIP, vasoactive intestinal peptide
